# Supramolecular Assembly of Gold Nanoparticles on Carbon Nanotubes: Application to the Catalytic Oxidation of Hydroxylamines

**DOI:** 10.3390/nano6030037

**Published:** 2016-02-24

**Authors:** Nimesh Shah, Pallabita Basu, Praveen Prakash, Simon Donck, Edmond Gravel, Irishi N. N. Namboothiri, Eric Doris

**Affiliations:** 1 Department of Chemistry, Indian Institute of Technology, Bombay, Mumbai 400076, India; nimesh31@yahoo.com (N.S.); pallabita17@gmail.com (P.B.); simon.donck@gmail.com (S.D.); 2 Alternative Energies and Atomic Energy Commission (CEA), Saclay Institute of Biology and Technology (IBITECS), Department of Bioorganic Chemistry and Isotopic Labeling, 91191 Gif-sur-Yvette, France; praveen.prakash@cea.fr (P.P.); edmond.gravel@cea.fr (E.G.)

**Keywords:** carbon nanotubes, gold nanoparticles, nanohybrid, heterogeneous catalysis

## Abstract

A supramolecular heterogeneous catalyst was developed by assembly and stabilization of gold nanoparticles on the surface of carbon nanotubes. A layer-by-layer assembly strategy was used and the resulting nanohybrid was involved in the catalytic oxidation of hydroxylamines under mild conditions. The nanohybrid demonstrated high efficiency and selectivity on hydroxylamine substrates.

## 1. Introduction

Besides stoichiometric approaches that are routinely used for the oxidation of organic substrates [1,2], catalytic processes have also been devised to perform selective oxidation reactions [3]. With heterogeneous catalytic systems, obtained by assembling the metallic catalysts on a solid support, facile reclaim and reuse of the catalytic species can be achieved [4]. Among the various platforms used as supports, allotrope forms of carbon, in particular carbon nanotubes (CNTs), have been shown to provide some key advantages [5]. The latter include chemical, thermal, and mechanical stability, high specific surface area, inertness, and adjustable topography. CNTs are also able to act in a synergistic fashion to enhance the performances of the supported catalytic metal [6,7]. With these features in mind, we sought to develop a catalyst that could catalyze some oxidation reactions under mild conditions of temperature and pressure using low catalytic loadings. Herein, we report the assembly of a CNT-gold nanohybrid catalyst and its application to the selective oxidation of hydroxylamines into either nitroso or azoxy derivatives.

## 2. Results and Discussion

### 2.1. Assembly of the AuCNT Nanohybrid

**Figure 1:**
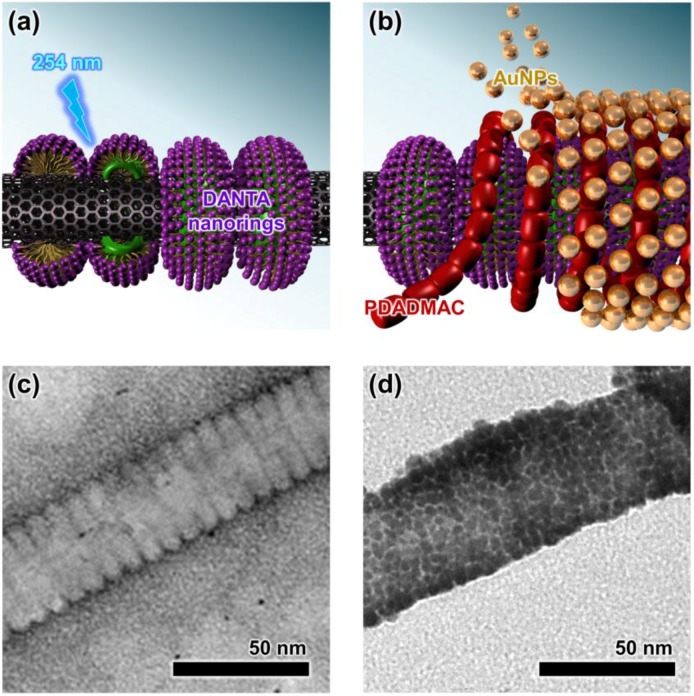
(**a**) Schematic representation of nitrilotriacetic-diyne (DANTA) nanoring polymerization on the carbon nanotube (CNT) surface; (**b**) Schematic representation of the final stages of the AuCNT synthesis; (**c**) Transmission electron microscopy (TEM) image of polymerized DANTA nanorings on CNT (negative staining); (**d**) TEM image of the AuCNT final catalyst.

Although gold has long been regarded as a poor catalytic metal, its nanosized forms [8], including supported ones [9,10], have recently been shown to be able to catalyze a wide array of chemical transformations. Our CNT-gold catalyst was built using a layer-by-layer approach that was adapted from our previous work [11,12,13,14,15,16,17,18,19,20,21,22,23]. Carbon nanotubes were first dispersed in an aqueous solution by ultrasonication in the presence of an amphiphilic nitrilotriacetic-diyne (DANTA) surfactant. This first step led to the non-stochastic assembly of the amphiphilic units on the CNT surface and to the formation of supramolecular structures with a nanoring-like shape (Figure 1a,c). This type of well-ordered supramolecular assembly on CNTs was first reported in 2003 [24]. Amphiphilic DANTA adsorbed at the surface of the nanotubes by hydrophobic interactions while its hydrophilic polar head was pointing toward the aqueous medium. The rings were polymerized by ultraviolet (UV) irradiation in a second step. In fact, irradiation of the sample for 6 h at 254 nm led to a topochemical polymerization of the diyne motif incorporated in the hydrophobic part of the starting amphiphile [25,26]. Polymerization takes place within individual half-cylinders and strengthens the cohesion of the assembly. After UV irradiation, the DANTA-decorated nanotubes became resistant to dialysis against water and to ethanol washes, indicating that the lipid assemblies had been polymerized. The second layer was thereafter deposited by stirring the suspended nanotubes with a cationic polymer, poly(diallyldimethylammonium chloride) (PDADMAC), which adsorbed on the nanotube’s surface by electrostatic interactions with the primary anionic layer. The double-coated CNTs were then recovered by centrifugation before the final deposition of gold nanoparticles (AuNPs). The latter were prepared in parallel by reduction of HAuCl_4_ in the presence of tetrahydroxymethylphosphonium chloride [27]. The colloidal suspension of gold nanoparticles (AuNPs) was then sequentially added to the coated CNTs in which the polyammonium network provided robust anchoring and stabilization of the metallic nanoparticles (Figure 1b,d). The AuCNT nanohybrid was finally suspended in water and used for the catalysis of the reported reactions.

### 2.2. Characterization of the AuCNT Nanohybrid

**Figure 2:**
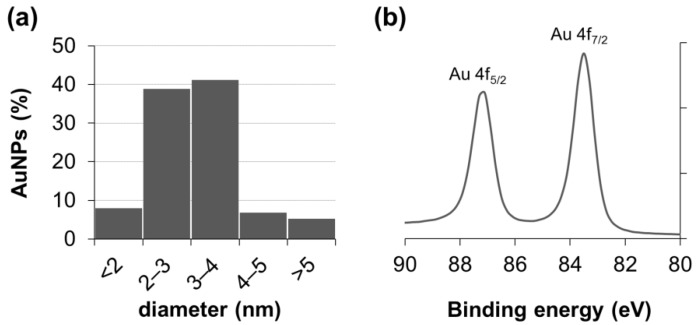
(**a**) Size distribution obtained from the measurement of 250 gold particles; (**b**) X-ray photoelectron spectroscopy (XPS) analysis (Au 4f core level) of the AuCNT nanohybrid.

Transmission electron microscopy (TEM, Philips CM12 microscope, Amsterdam, the Netherlands) indicated that the AuNPs were of spherical shape. The average size of the supported nanoparticles was measured using TEM pictures. Statistical measurement indicated that the supported AuNPs had an average diameter of *ca.* 3 nm (Figure 2a). The metal content of the aqueous AuCNT suspension was determined by inductively coupled plasma mass spectrometry (ICP-MS) ([Au] = 1.2 mM). Finally, the metallic character of the supported gold nanoparticles was established by X-ray photoelectron spectroscopy (XPS, VG ESCALAB 210 spectrometer, Waltham, MA, USA) analysis which showed characteristic Au 4f-binding energies contributions of Au [28] (Figure 2b).

### 2.3. Oxidation of Hydroxylamines with the AuCNT Nanohybrid

With the gold-based nanohybrid in hand, we investigated its potential in the aerobic oxidation of hydroxylamines. In the latter transformation, we expected the formation of the corresponding nitroso derivatives [29]. The nanohybrid-catalyzed oxidation of *tert*-butyl hydroxylamine (**1a**) in CHCl_3_/H_2_O afforded *tert*-butyl nitroso compound **1b** in 81% yield after 12 h of reaction (Table 1, Entry 1). The use of a binary solvent mixture permitted us to increase the rate of the reaction and afforded a more selective transformation. The reaction of *N*-cyclohexylhydroxylamine (**2a**) also cleanly produced nitrosocyclohexane (**2b**) in 83% yield (Entry 2). It is noteworthy that no isomerization of nitrosocyclohexane (**2b**) into the corresponding cyclohexanone oxime was detected. This result is to be noted since tautomerization of nitroso compounds to oximes is classically observed when working with substrates carrying Cα-protons. The reaction of aliphatic substrates thus satisfactorily provided access to nitroso compounds in high yields, but aromatic hydroxylamines did not behave similarly.

**Scheme 1:**
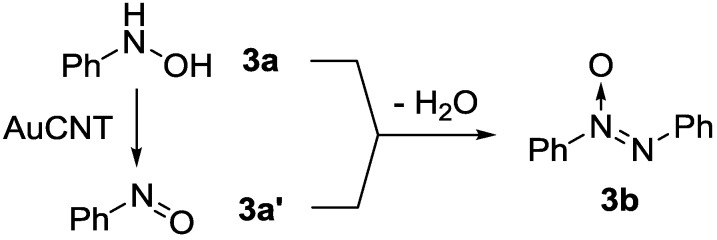
Postulated mechanism for the formation of azoxy compounds.

For example, the reaction of phenylhydroxylamine (**3a**) with AuCNT in CHCl_3_ gave a dimeric product in the form of the azoxy derivative **3b** in 2 h only (Entry 3). It is noteworthy that the binary solvent system was not required in the case of aromatic hydroxylamine substrates. A possible mechanism for the conversion observed in entry 3 (formation of the azoxy compound) could involve the oxidation of phenylhydroxylamine (**3a**) into the corresponding nitroso derivative **3a’** (Scheme 1). As **3a’** accumulates in the solution, its condensation with the unreacted phenylhydroxylamine (**3a**) leads to the formation of azoxy derivative **3b**, after the elimination of a molecule of water.

The same type of reactivity was also evidenced for other aromatic substrates such as 4-chlorophenylhydroxylamine (**4a**, Entry 4) and 4-fluorophenylhydroxylamine (**5a**, Entry 5). Indeed, we detected the formation of azoxy dimers **4b** (98% yield) and **5b** (92% yield) starting from compounds **4a** and **5a**, respectively. The transformation worked equally well on benzylhydroxylamine (**6a**) in 94% yield (Entry 6) but failed on the more hindered 2,6-dimethylphenylhydroxylamine (**7a**, Entry 7) which remained unaffected.

#### 2.3.1. Recycling of the AuCNT Nanohybrid

To assess the recyclability of the AuCNT nanohybrid, multiple oxidation cycles were carried out by successive reuse of the same sample of catalyst. A classical oxidation reaction was set using the general procedure described above which was applied to *N*-phenylhydroxylamine (**3a**). After completion, the catalyst was recovered by centrifugation, and the supernatant was worked up. The catalyst was washed with tetrahydrofuran (THF) and reused in subsequent oxidation reactions. This process was repeated over five consecutive cycles and showed no significant decrease in yields of oxidized azo product **3b** (Table 2). After the fifth run, TEM analysis showed no major alteration of the nanohybrid morphology. The use of non-supported AuNPs provided lower yields of product and the catalyst could not be recycled.

#### 2.3.2. Is AuCNT a Heterogeneous Catalyst?

The involvement of the nanohybrid in the oxidation process was demonstrated by the following experiment. A standard oxidation reaction of *N*-phenylhydroxylamine (**3a**) was run and, after 45 min, split into two. At this stage, approximately 50% conversion of **3a** into the corresponding azoxy derivative **3b** was detected by ^1^H-NMR (Bruker Avance DPX 400 MHz spectrometer, Billerica, MA, USA). The AuCNT catalyst was removed by centrifugation in one sample, whereas it was left in the other. Both reactions were further stirred for additional time before analysis. While the reaction was nearly quantitative (*ca.* 95% yield) in the AuCNT-containing sample, no further conversion was detected in the absence of the nanohybrid. These observations confirmed that oxidation of *N*-phenylhydroxylamine (**3a**) occurred at the surface of the AuCNT nanohybrid which acted as a solid catalyst [30] and whose performances are comparable to that of a previously developed rhodium-carbon nanotube catalytic system [23].

## 3. Experimental Section

### 3.1. Assembly of the Nanohybrid

Amphiphilic DANTA (20 mg) was dissolved in 2 mL of 25 mM pH 8 aqueous Tris-buffer before multiwalled carbon nanotubes (50 mg) were added. The dispersion was sonicated and the stable suspension transferred into two tubes. The tubes were centrifuged at 5000× *g* and the supernatants were collected. The latter were centrifuged at 15,000× *g* for 45 min. The supernatant was discarded and the pellets taken in buffer and centrifuged again at 15,000× *g* for 45 min. The pellets were finally resuspended in 1.5 mL of buffer and submitted to UV irradiation at 254 nm for 6 h.

After polymerization, the buffer volume was adjusted to 1.5 mL. The suspension was stirred in the presence of the cationic polymer PDADMAC (700 µL of a 20% water solution) for 1 h. The ensuing centrifugation at 15,000× *g* for 30 min permitted to get rid of the polymer in excess. The pellets were taken in 2 mL of buffer. This operation was repeated twice using the buffer solution and two more times using pure water.

The final pellets were resuspended in 1 mL of water. Then 50 μL of the latter suspension was transferred to Eppendorf^®^ tubes (×20). To each tube was added 1 mL of a 1 mM colloid suspension of the gold nanoparticles [27] and the mixture was vortex-stirred at room temperature for 1 min every 30 min (during 4 h). The suspension was then centrifuged at 3000× *g* for 5 min. The supernatant was discarded and 1 mL of a fresh gold colloid suspension was added. The same process was repeated two more times. The pellets were washed three times by centrifugation/redispersion in water. The 20 pellets were combined and 4 mL of water was finally added.

### 3.2. Procedure for the Oxidation of Hydroxylamines

A typical procedure is given for the oxidation of *N*-cyclohexylhydroxylamine **2a**. Under air, to a stirred solution of *N*-cyclohexylhydroxylamine hydrochloride (**2a**.HCl, 0.1 mmol) in 2 mL of CHCl_3_/H_2_O (1:1) was added K_2_CO_3_ (2 equivalents) and 0.5 mol % of the suspension of the AuCNT catalyst. The reaction mixture was stirred at room temperature for 12 h. The aqueous layer was extracted with CHCl_3_. The combined organic layer was dried over anhydrous Na_2_SO_4_, filtered, and concentrated under vacuum. The crude residue was purified by column chromatography to afford nitroso-cyclohexyl amine **2b** in 83% yield. ^1^H-NMR (400 MHz, CDCl_3_) δ (ppm) 5.07 (triplet of triplet, *J* = 3.8 Hz, *J* = 11.6 Hz, 1H), 1.97–1.94 (multiplet, 2H), 1.89–1.86 (multiplet, 2H), 1.69–1.65 (multiplet, 2H), 1.43–1.31 (multiplet, 2H), 1.28–1.20 (multiplet, 2H); ^13^C-NMR (100 MHz, CDCl_3_) δ (ppm) 65.6, 28.2 (2C), 25.0, 24.5 (2C).

## 4. Conclusions

A nanohybrid catalyst was produced by the layer-by-layer supramolecular assembly of gold nanoparticles on carbon nanotubes. The gold-based nanohybrid (AuCNT) was employed in the oxidation of hydroxylamines to provide straightforward access to the corresponding oxidized products in good to excellent yields. The transformation led either to nitroso derivatives in the case of aliphatic hydroxylamines or azoxy derivatives in the case of aromatic/benzylic hydroxylamines. Selectivity, low catalyst loading and mild reaction conditions (e.g., room temperature, open air) are the salient features of the AuCNT methodology.

## Figures and Tables

**Table 1:** Oxidation of various hydroxylamines with AuCNT ^[a]^. 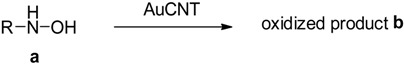

Entry	Substrate a	Product b	Yield (%)
1	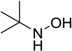	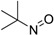	81 ^[b]^
2	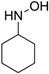	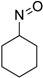	83 ^[b]^
3	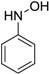	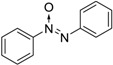	96 ^[c]^
4	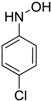	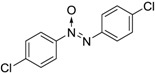	98 ^[c]^
5	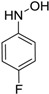	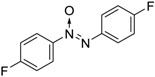	92 ^[c]^
6	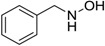	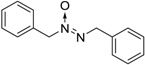	94 ^[c]^
7	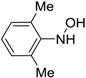	NR	-- ^[c]^

[a] Conditions: *N*-hydroxylamine (0.1 mmol), AuCNT (0.5 mol %), K_2_CO_3_ (0.2 mmol), room temperature under air. [b] Reaction time 12 h, CHCl_3_/H_2_O (1:1, 2 mL). [c] Reaction time 2 h, CHCl_3_ (2 mL).

**Table 2:** Recycling of AuCNT for the oxidation of *N*-phenylhydroxylamine. 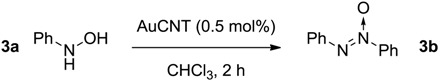

Entry	AuCNT	Yield (%)
1	fresh	96
2	1st reuse	93
3	2nd reuse	91
4	3rd reuse	93
5	4th reuse	94
